# Quantifying the Levels of Knowledge, Attitude, and Practice Associated with Sickle Cell Disease and Premarital Genetic Counseling in 350 Saudi Adults

**DOI:** 10.1155/2019/3961201

**Published:** 2019-05-02

**Authors:** Heba M. Al-Qattan, Dana F. Amlih, Fatima S. Sirajuddin, Dalal I. Alhuzaimi, Mai S. Alageel, Reema M. Bin Tuwaim, Farjah H. Al Qahtani

**Affiliations:** ^1^College of Medicine, King Saud University, Riyadh 11451, Saudi Arabia; ^2^Division of Oncology, Department of Medicine, College of Medicine, King Saud University, Riyadh, Saudi Arabia

## Abstract

Our study aims to observe the levels of knowledge, attitude, and practice (KAP) associated with sickle cell disease (SCD) and premarital genetic counseling (PMGC) in 351 Saudi adults. The relationships between KAP levels and sociodemographic characteristics (age, gender, marital status, and educational level) were observed. The study was conducted in King Khalid University Hospital between February 21, 2017, and March 7, 2018. A total of 351 Saudi participants attending the primary care clinic were selected using convenience sampling and were given a self-administered questionnaire. Overall, the 351 participants had the best attitude (41% scoring “good”), followed by knowledge (28.8%), and, lastly, practice (19.1%). Out of the sociodemographic characteristics, age group was the most statistically significant in all the three categories (knowledge, attitude, and practice). The > 50-year age group performed the worst in all the three categories. Despite the advancements in public healthcare measures in Saudi Arabia, our study revealed that there are still many gaps to be filled regarding the knowledge, attitude, and practice associated with SCD and PMGC.

## 1. Introduction

Sickle cell disease (SCD) is an inherited autosomal recessive blood disorder that causes red blood cells to become rigid and crescent-shaped [1]. This leads to several complications, including hand-foot syndrome, recurrent infections, delayed growth, vision problems, vasoocclusion, chronic hemolysis, acute and chronic kidney disease, and, eventually, progressive multiorgan damage [1–7] and stroke [8]. SCD sufferers also have decreased life expectancy and low quality of life [3, 7–10].

SCD is one of the most widespread monogenic diseases in the world, with over 300,000 babies born with SCD every year [4, 11]. It commonly affects many African and Asian countries, with the Middle East being one of the most prominently affected regions [1, 4, 12–14]. In Saudi Arabia, studies show that SCD is a relatively common genetic disorder. Up to 27% of the population have the trait, with 2.6%–4.2% of which being manifested as SCD [3, 13, 15]. The Eastern province has the highest prevalence (145 cases/10,000 population), followed by southwestern provinces (24 cases/10,000 population) [3, 10, 16, 17]. High SCD prevalence in Saudi Arabia is due to the high occurrence of consanguinity between first cousins (> 50% of total marriages) [12–14, 18, 19] and the population's lack of awareness of inherited hematological diseases [14]. Additionally, SCD carriers are resistant to Falciparum malaria, which is endemic in the region. This heterozygote advantage also contributes to the increased prevalence of SCD in Arab countries [12, 13].

Recent studies show no significant changes in SCD prevalence [1, 3, 9, 18, 20]. Previous studies have proven that, despite the legal implementation of compulsory premarital genetic counseling (PMGC), the incidence of SCD in Saudi Arabia has not changed significantly over the last 15 years [1, 9, 18, 20]. The lack of KAP regarding SCD caused an increase in the disease incidence and a decrease in the quality of life among the disease sufferers [1, 3, 9, 18, 20].

SCD still remains one of the biggest unspoken issues Saudi Arabia faces today, with one of the highest prevalence rates worldwide. Studies show that this is no coincidence, as SCD has been directly linked to consanguinity, a common practice in Saudi Arabia. Unlike most other diseases, cultural stigma proved to be a direct cause of high SCD incidence rates. This is a unique aspect of SCD KAP studies that requires further exploration so that suitable and effective prevention measures can be taken.

## 2. Materials and Methods

A cross-sectional KAP study was conducted in King Khalid University Hospital (KKUH) between February 21, 2017, and March 7, 2018. A total of 351 Saudi participants attending the primary care clinic (PCC) in KKUH were selected using convenience sampling. The sample size was calculated using the standard single proportion formula, where the proportion (p* = *0.253) was obtained from a previous similar study [1]. After plugging in the values, the effective sample size was *n* = 290. An additional 60 participants (20% of the effective sample size) were added in case of invalid questionnaires, with the total sample size being 351 participants.

### 2.1. Inclusion Criteria

Saudi males and females of all educational levels, married or unmarried, over the age of 18 attending the PCC in KKUH between February 21, 2017, and March 7, 2018, who were able to sign the written informed consent and able to understand either Arabic or English were included in the study.

### 2.2. Exclusion Criteria

Participants who were under the age of 18, unable to sign the written informed consent, and unable to understand English or Arabic were excluded from the study. To avoid selection bias, known cases and/or family history of sickle cell disease, sickle cell trait, or any hereditary hematological diseases were also excluded from the study.

### 2.3. Questionnaire

After the participants provided informed consent, a self-administered, validated questionnaire was given to each participant. The KAP questionnaire was obtained from two similar previous studies with the authors' permissions [14, 21]. Questions 1–10 asked about sociodemographic data. The rest of the questionnaire was used for KAP assessment, where 1 point was given for every correct answer. The knowledge section assessed the participant's awareness of SCD and PMGC. It contained 9 questions and, hence, the maximum score a participant could obtain in this section was 9 points (*good knowledge,* 9–5 points;* poor knowledge,* 4–0 points). The attitude section assessed the participant's beliefs regarding PMGC. The maximum score a participant could obtain in this section was 6 points (*good attitude,* 6–4 points;* poor attitude,* 3–0 points).

The practice section assessed the participant's implementation of PMGC in his/her life. The maximum score a participant could obtain in this section was 3 points (g*ood practice,* 3–2 points; p*oor practice,* 1–0 points). Data collection was performed with the help of trained volunteers. The questionnaire was cross-translated from Arabic to English and back to Arabic. The questionnaire was available in both Arabic and English and contained a paragraph for consent and other ethical considerations. For the illiterate group, the questionnaire and informed consent were read and explained to them by the trained volunteers.

### 2.4. Ethical Considerations

The study was approved by the King Saud University Institutional Review Board. All participants received a full written informed consent form in English or Arabic. Details informing the participants about the purpose of the research, why they were chosen, all potential risks and benefits, and that they could refuse to participate or withdraw from the study at any point in time were also provided at the beginning of the questionnaire in Arabic or English. The participants' identities were kept anonymous. No coercion, incentives, or rewards were used for the participants who did not wish to participate. Private and/or personal information was not and will not be disclosed during or after the study.

### 2.5. Statistical Methods of Analysis

The data were analyzed using Statistical Package for the Social Sciences (SPSS) Pc + 21.0 version statistical software. Descriptive statistics (frequencies and percentages) were used to describe the participants' sociodemographic characteristics (Table 1). Each questionnaire was graded manually to obtain 3 scores (knowledge score, attitude score, and practice score). Based on these scores, the frequencies of good and poor KAP were obtained using descriptive statistics (frequencies and percentages). Bivariate analysis between the frequencies of each sociodemographic characteristic and the levels of KAP (good/poor) was performed using crosstabulation (descriptive statistics) (Tables 2–4). Using Pearson chi-squared test, a p-value ≤ 0.05 and 95% confidence intervals were used to report the statistical significance and precision of results.

## 3. Results

A total of 351 Saudi participants were observed for SCD and PMGC KAP assessment. The participants were taken from both male and female PCCs in KKUH and came from all educational levels. Overall, the 351 participants had the best attitude (41% scoring “good”), followed by knowledge (28.8%), and, lastly, practice (19.1%). The most statistically significant association with all three outcome variables was age group, with the > 50-year age group performing the worst in all three categories. Correlation studies between knowledge, attitude, and practice showed significant correlations between attitude and knowledge (*p = 0.002)*, practice and knowledge (*p = 0.002)*, and practice and attitude* (p = 0.0001)*.

### 3.1. Knowledge

Out of the 351 participants, 101 (28.8%) had good knowledge (9–5 points out of 9) about SCD and premarital screening, while the remaining 250 participants (71.2%) had poor knowledge. The median score was 6 (interquartile range [IQR] = 2). A total of 237 (67.5%) participants had heard about SCD, but only 75 (21.4%) and 52 (14.8%) participants were aware of its treatment limitations and inheritance pattern, respectively. A total of 345 (98.3%) participants heard of PMGC, but 153 (43.6%) did not know the specific tests performed and diseases screened. When asked about their main source of knowledge regarding PMGC, 132 study participants (49.4%) chose friends and colleagues, while only 27 (10.1%) and 18 (6.7%) participants chose healthcare professionals and awareness programs, respectively. A total of 330 (94%) participants were aware that PMGC limits the spread of inherited blood disorders, and 268 (76.4%) participants were aware that families suffer from psychological stress if a member has an inherited blood disorder. Bivariate analysis showed significant (*p = 0.043*) association between the level of knowledge and age groups. The age group with best knowledge was 29–39 years, with 40 out of 112 (35.7%) participants scoring “good” for knowledge. The worst was > 50 years, with only 8 out of 48 (16.6%) participants scoring “good” for knowledge (Table 2). The other study variables showed no significant associations.

### 3.2. Attitude

Out of the 351 participants, 144 (41%) had good attitude (6–4 points out of 6), while the remaining 207 participants (59%) had poor attitude toward PMGC. The median score was 5 (IQR* = *1). Of the 268 married or previously married participants, 118 (44%) marriages were consanguineous. However, 69 (83.1%) of the 83 single participants did not prefer consanguineous marriages. A total of 291 (82.9%) of the 351 participants claimed that positive premarital test results would affect their decision to marry, and 336 (95.7%) and 341 (97.2%) participants supported the idea of compulsory premarital counseling and believed that it is important, respectively. In total, 347 (98.8%) participants would recommend premarital screening to others. Bivariate analysis showed significant (*p = 0.001*) association between attitude and educational levels. The educational level with the best attitude score was read/write followed by university, with 5 out of 9 (55.5%) and 104 out of 213 (48.8%) participants scoring “good” for attitude, respectively. The worst was the illiterate group, with only 1 out of 10 (10%) scoring “good” for attitude. There was also a significant (*p = 0.043)* association between the level of attitude and age groups. The age group with the best attitude was 18–28 years, with 52 out of the 113 (46%) participants scoring “good” for attitude. The worst was > 50 years, with only 11 out of 48 (22.9%) participants scoring “good” (Table 3). The other study variables showed no significant associations.

### 3.3. Practice

Out of the 351 participants, 67 (19.1%) had good practice (3–2 points out of 3), while the remaining 284 participants (80.9%) had poor practice toward premarital screening. The median score was 2 (IQR* =* 1). Out of the 268 married or previously married participants, only 147 (54.8%) participants performed premarital screening. However, of the remaining 83 single participants, 76 (91.5%) will do premarital screening in the future. When asked about how often the participants have been approached about SCD, 92 (26.2%), 137 (39%), and 122 (34.8%) chose* often*,* not often*, and* never*, respectively. When asked about the barriers faced in counseling for consanguinity, 113 (32.2%), 11 (3.1%), and 39 (11.1%) selected* knowledge*,* language, *and* both*, respectively. The remaining 188 (53.6%) participants reported to have no barriers. A total of 155 (44.1%) participants did not think they needed more education about genetic counseling. Bivariate analysis showed significant (*p = 0.014)* association between the practice levels and age groups. The age group with the best practice was 29–39 years, with 31 out of the 112 (27.6%) participants scoring “good” for practice. The worst was > 50 years, with only 5 out of the 48 (10.4%) participants scoring “good” (Table 4). The other study variables showed no significant associations.

## 4. Discussion

Based on the results observed, it is clear that, despite PMGC being compulsory in Saudi Arabia, many still lack good KAP regarding PMGC and SCD. Numerous gaps regarding all the three aspects of the study were analyzed.

### 4.1. Knowledge

The knowledge level of the participants in our study (28.8% participants having good knowledge) was inconsistent with the other studies done in Bahrain (93%) [19], Oman (96%) [14], and Saudi Arabia (94.3%) [14, 22], but was similar to the results in Nigeria (17.8%) [1] and Sudan (26.9%) [2]. There was a large discrepancy in knowledge between our study (28.8%) and El-Hazmi's study (94.3%). This could be because El-Hamzi's participants were attendees of conferences, symposia, and awareness lectures [22], while our study participants were members of the general Saudi population. In our study, the fact that many knew what PMGC was but did not know what it screens for implied a lack of awareness and concern for such inherited diseases. When crosstabulation was performed for the screening tests and age groups, poor knowledge was demonstrated equally in all four age groups with no statistical significance. Moreover, many obtained their knowledge about PMGC clinics through friends and colleagues rather than through healthcare facilities. This could indicate a need for more PMGC awareness campaigns in healthcare institutions. Moreover, only 10.1% of the participants obtained their knowledge of PMGC clinics from healthcare workers. This reflects that health promotion in personal matters such as marriage may not be appropriate in Saudi culture. Therefore, the attitude of healthcare workers toward PMGC promotion needs further exploration. The cause of poor SCD knowledge among the Saudi population is not only a deficiency in seeking knowledge but also a deficiency in receiving knowledge from healthcare workers.

### 4.2. Attitude

Our attitude results (41% participants having good attitude) were similar to those of the previous studies in Saudi Arabia, with only 42% of the participants in Riyadh and 29% in Abha supporting the implementation of the compulsory PMGC law [14]. In Sudan, only 48% of the participants had adequate attitude [2]. However, other studies showed 86.9% and 70% of the participants supporting compulsory PMGC, respectively [14]. In our study, the participants performed the best in attitude out of all the three categories, which may indicate a shift in perspective toward consanguineous marriages. In fact, despite the high prevalence of consanguineous marriages in Saudi Arabia (51%–60%) [12–14, 18], most of the single participants in our study did not prefer consanguineous marriages. This was significantly associated with a younger age group (18–28 years) when crosstabulation was performed (*p = 0.0001*). This could mean a decrease in consanguineous marriages and inherited diseases in the future. When asked about the reason for not preferring consanguineous marriages, 51 out of the 69 single participants mentioned inherited diseases in their answers, implying that there could be a significant association between the increase in knowledge and the decrease in consanguineous marriages among young, single Saudi individuals. However, previous studies showed up to 90% of couples proceeding with marriage despite having a positive trait for a hematological disorder, mostly due to noncancellable wedding plans (43%–52%) and social stigma (21%) [14, 20, 23–25]. This infers that many single individuals do not prefer consanguineous marriages, but, when faced with the situation, must proceed with the marriage.

### 4.3. Practice

Out of all the three aspects observed in our study, the participants performed the worst in practice (19.1% participants having good practice). Our study revealed that the majority of the study participants did not undergo PMGC despite it being compulsory. It is also worth mentioning that although most married and previously married participants did not undergo PMGC, many young, single participants said that they will undergo PMGC in the future. This could imply a shift in future trends regarding the prevalence of inherited diseases [3], which needs further study. The reasons for poor practice should also be explored. In our study, lack of knowledge was the main reason for poor practice. This could be due to the barrier between healthcare workers and patients regarding sensitive topics such as marriage. Poor knowledge being the main cause of poor practice was consistent with other studies, along with misinterpretations of Islamic law [14, 23, 26], which was not explored in our study. Language was another main barrier among the study participants, similar to that seen among Dominicans in a study done in Manhattan [27]. Poor practice among the study participants may also indicate the lack of law regulation and governmental monitoring regarding PMGC.

### 4.4. Strengths

Our study adds more to the scarce literature [14]. The study participants were taken from the PCC in KKUH, which was representative of the Saudi population. Our study explored all three aspects of a KAP study, while most studies about PMGC solely focused on practice [14, 19, 23]. Age groups had high variability, which was a good representation of the Saudi population. The study had an adequate sample size with a high response rate. Open-ended questions were asked to find the root cause of the problem.

### 4.5. Limitations

Our study did not use random sampling, which created selection bias. The questionnaire did not contain an equal number of questions for KAP assessment, and practice questions were deficient. The study used “good” and “poor” for KAP assessment instead of “good,” “adequate,” and “poor.” There were fewer males (28.5%) than females (71.5%) who participated in the study and more married/previously married (76.4%) participants than previously unmarried (23.6%) participants, which was not representative of the population. Known cases and/or family history of SCD, sickle cell trait, or any other hereditary hematological diseases were excluded from our study. This limited the potential to compare results between participants with personal/family history of hereditary hematological diseases and participants without.

## 5. Conclusion

Despite the advancements in public healthcare measures in Saudi Arabia, our study revealed that there are still many gaps to be filled regarding the knowledge, attitude, and practice associated with SCD and PMGC. The challenges that Saudi Arabia faces for SCD control and prevention are numerous [3], and the implementation of PMGC law has not shown benefit in some studies [1, 3, 9, 18, 20]. Exploring these complex challenges in detail will help create an efficient and cost-effective plan for SCD prevention.

## Figures and Tables

**Table 1:** Sociodemographic characteristics of the participants.

*Variable*	*N (*%)
*Age* (years)	
18 – 28	113 (32.2)
29 – 39	112 (31.9)
40 – 50	78 (22.2)
> 50	48 (13.7)

*Gender* (Male)	100 (28.5)

*Marital status*	
Single	83 (23.6)
Married	240 (68.4)
Widowed	13 (3.7)
Divorced	15 (4.3)

*Educational level*	
Illiterate	10 (2.8)
Read/Write	9 (2.6)
Elementary	20 (5.7)
Intermediate	23 (6.6)
High School	76 (21.7)
University	213 (60.7)

**Table 2:** Level of knowledge in association with study variables.

*Study Variables*		*Knowledge N (*%)		*χ* ^*2*^ * value*	*P value*
		*Good*	*Poor*		
*Age (years)*	18 – 28	27 (26.7)	86 (34.4)	8.170	0.043
	29 – 39	40 (39.6)	72 (28.8)		
	40 – 50	26 (25.7)	52 (20.8)		
	>50 y	8 (7.9)	40 (16)		

*Gender*	Male	4 (33.7)	66 (26.4)	1.863	0.172
	Female	67 (66.3)	184 (73.6)		

*Marital status*	Single	18 (17.8)	65 (26)	5.611	0.132
	Married	78 (77.2)	162 (64.8)		
	Widowed	3 (3)	10 (4)		
	Divorced	2 (2)	13 (5.2)		

*Educational level*	Illiterate	2 (2)	8 (3.2)	5.680	0.339
	Read/Write	3 (3)	6 (2.4)		
	Elementary	2 (2)	18 (7.2)		
	Intermediate	6 (5.9)	17 (6.8)		
	High School	27 (26.7)	49 (19.6)		
	University	61 (60.4)	152 (60.8)		

**Table 3:** Level of attitude in association with study variables.

*Study Variables*		*Attitude N (*%)		*χ* ^*2*^ * value*	*P value*
		*Good*	*Poor*		
*Age (years)*	18 – 28	52 (36.1)	61 (29.5)	8.147	0.043
	29 – 39	46 (31.9)	66 (31.9)		
	40 – 50	35 (24.3)	43 (20.8)		
	>50	11 (7.6)	37 (17.9)		

*Gender*	Male	35 (24.3)	65 (31.4)	2.099	0.147
	Female	109 (75.7)	142 (68.6)		

*Marital status*	Single	44 (30.6)	39 (18.8)	7.233	0.065
	Married	89 (61.8)	151 (72.9)		
	Widowed	4 (2.8)	9 (4.3)		
	Divorced	7 (4.9)	8 (3.9)		

*Educational level*	Illiterate	1 (0.7)	9 (4.3)	21.197	0.001
	Read/Write	5 (3.5)	4 (1.9)		
	Elementary	9 (6.3)	11 (5.3)		
	Intermediate	4 (2.8)	19 (9.2)		
	High School	21 (14.6)	55 (26.6)		
	University	104 (72.2)	109 (52.7)		

**Table 4:** Level of practice in association with study variables.

*Study Variables*		*Practice N (*%)		*χ* ^*2*^ * value*	*P value*
		*Good*	*Poor*		
*Age (years)*	18 – 28	22 (32.8)	91 (32.0)	10.578	0.014
	29 – 39	31 (46.3)	81 (28.5)		
	40 – 50	9 (13.4)	69 (24.3)		
	>50	5 (7.5)	43 (15.1)		

*Gender*	Male	22 (32.8)	78 (27.5)	0.768	0.381
	Female	45 (67.2)	206 (72.5)		

*Marital status*	Single	10 (14.9)	73 (25.7)	4.063	0.255
	Married	51 (76.1)	189 (66.5)		
	Widowed	2 (3.0)	11 (3.9)		
	Divorced	4 (6.0)	11 (3.9)		

*Educational level*	Illiterate	3 (4.5)	7 (2.5)	3.217	0.667
	Read/Write	2 (3.0)	7 (2.5)		
	Elementary	2 (3.0)	18 (6.0)		
	Intermediate	6 (9.0)	17 (6.0)		
	High School	12 (17.9)	64 (22.5)		
	University	42 (62.7)	171 (60.2)		

## Data Availability

The data that support the findings of this study are available from the corresponding author, upon reasonable request.

## Additional Points

Future studies should focus on the expected decrease in inherited hematological diseases and its association with the decrease in consanguineous marriages. A KAP study focusing on single, young individuals in Saudi Arabia regarding SCD and PMGC should also be conducted to detect future trends. A KAP study comparing Riyadh to more rural areas in Saudi Arabia should be conducted, as rural areas tend to have different results than urban areas. Barriers of poor PMGC practice should be explored, especially regarding healthcare workers' attitudes toward PMGC promotion. Inconsistencies regarding consanguineous marriages must also be explored, as many single participants said that they did not prefer consanguineous marriages, despite its steady high prevalence. A study measuring the KAP level difference between participants with family history of SCD and participants without should also be conducted.
